# 1,4-Di-*tert*-butyl (2*R*,3*R*)-2-({(2*E*)-3-[4-(acet­yloxy)phen­yl]prop-2-eno­yl}­oxy)-3-hy­droxy­butane­dioate

**DOI:** 10.1107/S160053681200236X

**Published:** 2012-01-25

**Authors:** Josh L. Hixson, Dennis K. Taylor, Seik Weng Ng, Edward R. T. Tiekink

**Affiliations:** a School of Agriculture, Food and Wine, The University of Adelaide, Waite Campus, PMB 1, Glen Osmond, SA 5064, Australia; b Department of Chemistry, University of Malaya, 50603 Kuala Lumpur, Malaysia; c Chemistry Department, Faculty of Science, King Abdulaziz University, PO Box 80203 Jeddah, Saudi Arabia

## Abstract

The title compound, C_23_H_30_O_9_, has an approximate T-shape with the *tert*-butyl ester groups lying either side of the benzene ring. The acetyl group is almost perpendicular to the benzene ring to which it is connected [C—C—O—C torsion angle = −106.7 (3)°]. The conformation about the C=C double bond [1.331 (4) Å] is *E*. Linear supra­molecular chains along the *a* axis mediated by hy­droxy–carbonyl O—H⋯O hydrogen bonds feature in the crystal packing. The same H atom is also involved in an intra­molecular O—H⋯O inter­action.

## Related literature

For background to the formation of the odorant 4-ethyl­phenol with relevance to the wine industry, see: Chatonnet *et al.* (1992 ▶); Hixson *et al.* (2012 ▶); Ong & Nagel (1978 ▶); Nagel & Wulf (1979 ▶); Zhao & Burke (1998 ▶). For the preparation and characterization of 1-*O*-acetyl *p*-coumaric acid; see: Zhao & Burke (1998 ▶); Shimizu & Kojima (1984 ▶).
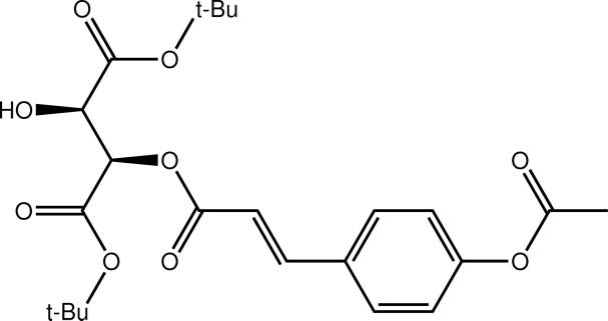


## Experimental

### 

#### Crystal data

C_23_H_30_O_9_
*M* *_r_* = 450.47
Orthorhombic, 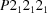

*a* = 5.7183 (2) Å
*b* = 8.7309 (3) Å
*c* = 46.9988 (19) Å
*V* = 2346.46 (15) Å^3^
*Z* = 4
Cu *K*α radiation
μ = 0.82 mm^−1^
*T* = 100 K
0.35 × 0.10 × 0.02 mm

#### Data collection

Agilent SuperNova Dual diffractometer with an Atlas detector
Absorption correction: multi-scan (*CrysAlis PRO*; Agilent, 2010 ▶) *T*_min_ = 0.230, *T*_max_ = 1.000
9078 measured reflections
4603 independent reflections
3842 reflections with *I* > 2σ(*I*)
*R*_int_ = 0.044

#### Refinement

*R*[*F* ^2^ > 2σ(*F* ^2^)] = 0.053
*wR*(*F* ^2^) = 0.131
*S* = 1.05
4603 reflections
300 parameters
H atoms treated by a mixture of independent and constrained refinement
Δρ_max_ = 0.28 e Å^−3^
Δρ_min_ = −0.30 e Å^−3^
Absolute structure: Flack (1983 ▶), 1849 Friedel pairs
Flack parameter: 0.0 (2)

### 

Data collection: *CrysAlis PRO* (Agilent, 2010 ▶); cell refinement: *CrysAlis PRO*; data reduction: *CrysAlis PRO*; program(s) used to solve structure: *SHELXS97* (Sheldrick, 2008 ▶); program(s) used to refine structure: *SHELXL97* (Sheldrick, 2008 ▶); molecular graphics: *ORTEP-3* (Farrugia, 1997 ▶) and *DIAMOND* (Brandenburg, 2006 ▶); software used to prepare material for publication: *publCIF* (Westrip, 2010 ▶).

## Supplementary Material

Crystal structure: contains datablock(s) global, I. DOI: 10.1107/S160053681200236X/hb6610sup1.cif

Structure factors: contains datablock(s) I. DOI: 10.1107/S160053681200236X/hb6610Isup2.hkl

Supplementary material file. DOI: 10.1107/S160053681200236X/hb6610Isup3.cml

Additional supplementary materials:  crystallographic information; 3D view; checkCIF report

## Figures and Tables

**Table 1:** Hydrogen-bond geometry (Å, °)

*D*—H⋯*A*	*D*—H	H⋯*A*	*D*⋯*A*	*D*—H⋯*A*
O16—H16⋯O27^i^	0.85 (4)	2.06 (4)	2.842 (3)	153 (4)
O16—H16⋯O30	0.85 (4)	2.26 (4)	2.688 (3)	111 (3)

Symmetry code: (i) 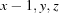.

**Fig. 1.**
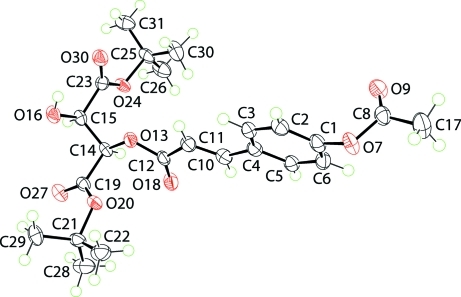
The asymmetric unit of (I) showing displacement ellipsoids at the 70% probability level.

**Fig. 2.**
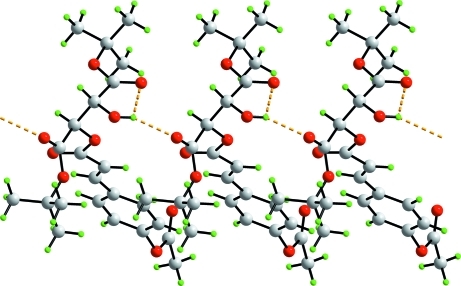
A view of the linear supramolecular chain along the a axis mediated by O—H···O hydrogen bonds shown as orange dashed lines.

## supplementary crystallographic information

### Comment

The breakdown of *p*-coumaric acid by *D. bruxellensis* to form the potent odorant 4-ethylphenol has been known for decades (Chatonnet *et al.*, 1992). Recently, it has been found that the metabolism of the ethyl ester of *p*-coumaric acid by this yeast can also result in the accumulation of significant concentrations of 4-ethylphenol (Hixson *et al.*, 2012). Existing in both the grape berry and in wine in significant concentrations (Ong & Nagel, 1978; Nagel & Wulf, 1979), the *p*-coumaroyl *L*-tartrate ester has the potential to contribute even further to the accumulation of 4-ethylphenol in finished wines and thus contribute further to the spoilage of wine.

Synthesis of the known wine component *p*-coumaroyl *L*-tartrate was attempted using di-*tert*-butyl *L*-tartrate and the 1-*O*-acetyl protected hydroxycinnamic acid in an analogous method to that described by Zhao and Burke (1998). The desired product was isolated and recrystallized from 30% ethyl acetate/hexane to afford a crystalline solid from which the structure was determined by X-ray crystallography to confirm the retention of the (*R*,*R*)-stereochemistry, Fig. 1.

The most prominent feature of the crystal packing is the formation of linear supramolecular chains along the *a* axis *via* hydroxyl-*O*—*H*···*O*(carbonyl) hydrogen bonds, Fig. 2 and Table 1. The hydroxyl-H atom is bifurcated, also forming an intramolecular O–H···O hydrogen bond with the adjacent carbonyl-O atom, Table 1.

### Experimental

1-*O*-Acetyl *p*-coumaric acid was prepared using a method analogous to that previously described by Zhao and Burke (1998), and the characterization data matched that previously described (Shimizu & Kojima, 1984). 1-*O*-Acetyl *p*-coumaric acid (0.17 g 0.82 mmol) was heated under reflux in dry benzene (10 ml) containing thionyl chloride (1 ml, 13.77 mmol). After 5 h the mixture was allowed to cool to room temperature and then concentrated *in vacuo*. The crude residue was taken up in dry benzene (3 ml) and added drop-wise to a solution of di-*tert*-butyl *L*-tartrate (0.13 g, 0.49 mmol) in dry pyridine (3 ml), then stirred at ambient temperature overnight. The mixture was concentrated and pyridine azeotropically removed with toluene. Purification with column chromatography (20% EtOAc/X4) and recrystallization from 30% EtOAc/X4 gave 68.1 mg (31%) of colourless plates. *M*.pt 416.8–417.4 K. *R*_f_ (50% EtOAc/X4): 0.57 ^1^H NMR: (400 MHz, CDCl_3_) δ: 7.73 (d, 1H, J = 16.0 Hz, H_7_), 7.54 (app. d, 2H, J = 8.7 Hz, H_3,5_), 7.13 (app. d, 2H, J = 8.7 Hz, H_2,6_), 6.45 (d, 1H, J = 16.0 Hz, H_8_), 5.48 (d, 1H, J = 2.3 Hz, H_2'_), 4.67 (dd, 1H, J = 6.9 and 2.3 Hz, H_3'_), 3.20 (d, 1H, J = 6.9 Hz, OH), 2.31 (s, 3H, OCOCH_3_), 1.51 (s, 9H, ^t^Bu_4_), 1.44 (s, 9H, ^t^Bu_1_). ^13^C NMR: (600 MHz, CDCl_3_) δ: 170.2 (C4'), 169.3 (OCOCH_3_), 165.8 (C9), 165.5 (C1'), 152.4 (C1), 145.5 (C7), 131.9 (C4), 129.6 (C3,5), 122.3 (C2,6), 116.7 (C8), 84.0 (C_1_(CH_3_)_3_), 83.4 (C_4_(CH_3_)_3_), 73.5 (C2'), 71.0 (C3'), 28.1 (C_4_(CH_3_)_3_), 28.0 (C1(CH_3_)_3_), 21.3 (OCOCH_3_). LRP (+EI) m/*z* (%): 450 (*M*+, <1), 408 (2), 352 (10), 338 (5), 321 (12), 296 (63), 278 (6), 251 (6), 206 (7), 189 (46), 164 (79), 147 (100), 119 (14), 57 (37), 41 (13). HRMS calculated for C_23_H_30_O_9_ [*M*]+ 450.1890, found 450.1891.

### Refinement

Carbon-bound H-atoms were placed in calculated positions [C—H 0.95–1.00 Å, *U*_iso_(H) 1.2–1.5*U*_eq_(C)] and were included in the refinement in the riding model approximation. The acid H-atom was located in a difference Fourier map and was refined freely.

### Crystal data

**Table:**

C_23_H_30_O_9_	*F*(000) = 960
*M**_r_* = 450.47	*D*_x_ = 1.275 Mg m^−^^3^
Orthorhombic, *P*2_1_2_1_2_1_	Cu *K*α radiation, λ = 1.54184 Å
Hall symbol: P 2ac 2ab	Cell parameters from 2558 reflections
*a* = 5.7183 (2) Å	θ = 2.8–74.4°
*b* = 8.7309 (3) Å	µ = 0.82 mm^−^^1^
*c* = 46.9988 (19) Å	*T* = 100 K
*V* = 2346.46 (15) Å^3^	Plate, colourless
*Z* = 4	0.35 × 0.10 × 0.02 mm

### Data collection

**Table:**

Agilent SuperNova Dual diffractometer with an Atlas detector	4603 independent reflections
Radiation source: SuperNova (Cu) X-ray Source	3842 reflections with *I* > 2σ(*I*)
Mirror	*R*_int_ = 0.044
Detector resolution: 10.4041 pixels mm^-1^	θ_max_ = 74.6°, θ_min_ = 3.8°
ω scan	*h* = −6→6
Absorption correction: multi-scan (CrysAlis PRO; Agilent, 2010)	*k* = −10→7
*T*_min_ = 0.230, *T*_max_ = 1.000	*l* = −56→58
9078 measured reflections	

### Refinement

**Table:**

Refinement on *F*^2^	Secondary atom site location: difference Fourier map
Least-squares matrix: full	Hydrogen site location: inferred from neighbouring sites
*R*[*F*^2^ > 2σ(*F*^2^)] = 0.053	H atoms treated by a mixture of independent and constrained refinement
*wR*(*F*^2^) = 0.131	*w* = 1/[σ^2^(*F*_o_^2^) + (0.0669*P*)^2^] where *P* = (*F*_o_^2^ + 2*F*_c_^2^)/3
*S* = 1.05	(Δ/σ)_max_ < 0.001
4603 reflections	Δρ_max_ = 0.28 e Å^−^^3^
300 parameters	Δρ_min_ = −0.30 e Å^−^^3^
0 restraints	Absolute structure: Flack (1983), 1849 Friedel pairs
Primary atom site location: structure-invariant direct methods	Flack parameter: 0.0 (2)

### Special details

**Table:**

Geometry. All s.u.'s (except the s.u. in the dihedral angle between two l.s. planes) are estimated using the full covariance matrix. The cell s.u.'s are taken into account individually in the estimation of s.u.'s in distances, angles and torsion angles; correlations between s.u.'s in cell parameters are only used when they are defined by crystal symmetry. An approximate (isotropic) treatment of cell s.u.'s is used for estimating s.u.'s involving l.s. planes.
Refinement. Refinement of *F*^2^ against ALL reflections. The weighted *R*-factor *wR* and goodness of fit *S* are based on *F*^2^, conventional *R*-factors *R* are based on *F*, with *F* set to zero for negative *F*^2^. The threshold expression of *F*^2^ > 2σ(*F*^2^) is used only for calculating *R*-factors(gt) *etc*. and is not relevant to the choice of reflections for refinement. *R*-factors based on *F*^2^ are statistically about twice as large as those based on *F*, and *R*- factors based on ALL data will be even larger.

### Fractional atomic coordinates and isotropic or equivalent isotropic displacement parameters (Å^2^)

**Table:**

	*x*	*y*	*z*	*U*_iso_*/*U*_eq_	
O7	−0.2022 (4)	0.5527 (2)	−0.00826 (4)	0.0253 (5)	
O9	−0.3426 (4)	0.3380 (2)	0.01232 (4)	0.0263 (4)	
O13	0.3891 (3)	0.9744 (2)	0.14782 (4)	0.0188 (4)	
O16	0.2916 (4)	1.1953 (2)	0.19343 (5)	0.0239 (4)	
H16	0.147 (7)	1.178 (5)	0.1910 (8)	0.042 (11)*	
O18	0.6765 (4)	0.8572 (2)	0.12326 (4)	0.0238 (4)	
O20	0.6536 (3)	1.2024 (2)	0.13224 (4)	0.0198 (4)	
O24	0.3937 (3)	0.8007 (2)	0.20890 (4)	0.0201 (4)	
O27	0.8203 (3)	1.2333 (2)	0.17558 (4)	0.0239 (4)	
O30	0.0551 (4)	0.9341 (2)	0.20280 (4)	0.0237 (5)	
C1	−0.0842 (5)	0.5915 (3)	0.01690 (6)	0.0223 (6)	
C2	−0.1778 (5)	0.7016 (3)	0.03504 (6)	0.0225 (6)	
H2	−0.3271	0.7450	0.0314	0.027*	
C3	−0.0493 (5)	0.7466 (3)	0.05852 (6)	0.0206 (6)	
H3	−0.1120	0.8210	0.0711	0.025*	
C4	0.1717 (5)	0.6843 (3)	0.06398 (6)	0.0199 (6)	
C5	0.2585 (5)	0.5716 (3)	0.04550 (6)	0.0211 (6)	
H5	0.4074	0.5272	0.0490	0.025*	
C6	0.1294 (5)	0.5244 (3)	0.02212 (6)	0.0230 (6)	
H6	0.1876	0.4467	0.0099	0.028*	
C8	−0.3134 (5)	0.4121 (3)	−0.00867 (6)	0.0221 (6)	
C10	0.3277 (5)	0.7401 (3)	0.08639 (6)	0.0211 (6)	
H10	0.4826	0.7008	0.0861	0.025*	
C11	0.2799 (5)	0.8390 (3)	0.10715 (6)	0.0203 (6)	
H11	0.1259	0.8775	0.1098	0.024*	
C12	0.4738 (5)	0.8874 (3)	0.12611 (6)	0.0185 (6)	
C14	0.5580 (5)	1.0245 (3)	0.16863 (6)	0.0167 (5)	
H14	0.6722	0.9400	0.1723	0.020*	
C15	0.4241 (5)	1.0593 (3)	0.19588 (6)	0.0183 (6)	
H15	0.5391	1.0723	0.2117	0.022*	
C17	−0.3823 (7)	0.3715 (3)	−0.03830 (7)	0.0339 (8)	
H17A	−0.5229	0.3073	−0.0379	0.051*	
H17B	−0.2546	0.3151	−0.0475	0.051*	
H17C	−0.4146	0.4652	−0.0491	0.051*	
C19	0.6905 (5)	1.1681 (3)	0.15925 (6)	0.0178 (5)	
C21	0.7821 (5)	1.3308 (3)	0.11844 (6)	0.0223 (6)	
C22	0.6898 (6)	1.3215 (4)	0.08806 (6)	0.0286 (6)	
H22A	0.5236	1.3489	0.0878	0.043*	
H22B	0.7091	1.2169	0.0809	0.043*	
H22C	0.7772	1.3927	0.0760	0.043*	
C23	0.2640 (5)	0.9244 (3)	0.20304 (6)	0.0174 (6)	
C25	0.2773 (5)	0.6517 (3)	0.21600 (6)	0.0231 (6)	
C26	0.4859 (5)	0.5481 (3)	0.22185 (7)	0.0296 (7)	
H26A	0.5811	0.5391	0.2046	0.044*	
H26B	0.4304	0.4464	0.2275	0.044*	
H26C	0.5806	0.5921	0.2372	0.044*	
C28	1.0416 (5)	1.3012 (4)	0.11923 (7)	0.0325 (7)	
H28A	1.0969	1.3064	0.1389	0.049*	
H28B	1.1226	1.3787	0.1078	0.049*	
H28C	1.0741	1.1992	0.1115	0.049*	
C29	0.7116 (6)	1.4816 (3)	0.13230 (7)	0.0274 (7)	
H29A	0.7685	1.4839	0.1520	0.041*	
H29B	0.5408	1.4908	0.1322	0.041*	
H29C	0.7801	1.5670	0.1216	0.041*	
C30	0.1389 (6)	0.5973 (4)	0.19021 (7)	0.0311 (7)	
H30A	0.2419	0.5935	0.1736	0.047*	
H30B	0.0099	0.6686	0.1865	0.047*	
H30C	0.0757	0.4949	0.1939	0.047*	
C31	0.1284 (5)	0.6724 (4)	0.24249 (7)	0.0296 (7)	
H31A	0.2228	0.7195	0.2576	0.044*	
H31B	0.0714	0.5724	0.2489	0.044*	
H31C	−0.0049	0.7388	0.2381	0.044*	

### Atomic displacement parameters (Å^2^)

**Table:**

	*U*^11^	*U*^22^	*U*^33^	*U*^12^	*U*^13^	*U*^23^
O7	0.0322 (11)	0.0166 (9)	0.0271 (11)	−0.0050 (9)	−0.0090 (9)	−0.0005 (8)
O9	0.0275 (11)	0.0233 (10)	0.0280 (11)	−0.0045 (9)	−0.0019 (9)	−0.0005 (8)
O13	0.0177 (9)	0.0169 (9)	0.0217 (10)	−0.0009 (8)	−0.0013 (8)	−0.0032 (7)
O16	0.0225 (11)	0.0137 (9)	0.0356 (12)	0.0024 (9)	0.0004 (9)	−0.0015 (8)
O18	0.0204 (11)	0.0193 (9)	0.0318 (11)	0.0008 (8)	−0.0023 (9)	−0.0052 (8)
O20	0.0217 (10)	0.0151 (9)	0.0226 (10)	−0.0043 (8)	0.0012 (8)	0.0019 (7)
O24	0.0179 (9)	0.0131 (9)	0.0293 (11)	−0.0016 (8)	0.0003 (8)	0.0048 (7)
O27	0.0244 (11)	0.0183 (9)	0.0290 (11)	−0.0032 (8)	−0.0056 (9)	0.0016 (8)
O30	0.0233 (11)	0.0180 (10)	0.0299 (12)	0.0001 (8)	0.0004 (9)	0.0000 (8)
C1	0.0289 (15)	0.0154 (12)	0.0225 (15)	−0.0040 (12)	−0.0026 (13)	0.0023 (11)
C2	0.0227 (14)	0.0180 (12)	0.0268 (14)	−0.0017 (12)	−0.0005 (12)	0.0021 (11)
C3	0.0221 (14)	0.0155 (12)	0.0242 (15)	−0.0023 (11)	0.0019 (11)	−0.0016 (11)
C4	0.0204 (14)	0.0167 (12)	0.0225 (13)	−0.0026 (11)	0.0012 (11)	0.0010 (10)
C5	0.0228 (14)	0.0158 (12)	0.0248 (14)	−0.0008 (11)	0.0022 (11)	0.0000 (10)
C6	0.0297 (16)	0.0175 (12)	0.0217 (14)	−0.0003 (12)	0.0018 (12)	−0.0023 (10)
C8	0.0221 (14)	0.0163 (12)	0.0277 (15)	0.0015 (11)	−0.0015 (13)	−0.0033 (11)
C10	0.0188 (14)	0.0196 (13)	0.0248 (14)	−0.0014 (11)	−0.0008 (12)	0.0012 (10)
C11	0.0187 (13)	0.0184 (13)	0.0238 (14)	−0.0015 (11)	−0.0008 (11)	−0.0007 (10)
C12	0.0197 (15)	0.0115 (12)	0.0242 (15)	−0.0023 (10)	0.0004 (11)	−0.0006 (10)
C14	0.0165 (13)	0.0119 (11)	0.0217 (14)	−0.0020 (10)	−0.0026 (11)	0.0007 (10)
C15	0.0206 (13)	0.0125 (12)	0.0218 (14)	0.0001 (11)	−0.0021 (11)	0.0002 (10)
C17	0.048 (2)	0.0223 (14)	0.0309 (17)	−0.0038 (14)	−0.0075 (16)	−0.0023 (13)
C19	0.0158 (13)	0.0135 (12)	0.0241 (14)	0.0003 (10)	−0.0005 (11)	0.0011 (10)
C21	0.0229 (14)	0.0152 (12)	0.0288 (15)	0.0001 (11)	0.0060 (12)	0.0056 (11)
C22	0.0310 (16)	0.0275 (15)	0.0274 (15)	0.0009 (13)	0.0054 (14)	0.0060 (12)
C23	0.0182 (14)	0.0176 (13)	0.0165 (13)	0.0003 (11)	−0.0006 (10)	−0.0006 (10)
C25	0.0247 (15)	0.0135 (12)	0.0311 (16)	−0.0033 (11)	−0.0002 (12)	0.0061 (11)
C26	0.0258 (15)	0.0207 (15)	0.042 (2)	0.0004 (12)	−0.0018 (13)	0.0086 (14)
C28	0.0271 (16)	0.0329 (17)	0.0375 (19)	0.0016 (14)	0.0079 (14)	0.0106 (15)
C29	0.0335 (17)	0.0134 (13)	0.0353 (17)	0.0033 (12)	−0.0024 (14)	0.0006 (11)
C30	0.0314 (17)	0.0219 (14)	0.0400 (18)	−0.0030 (13)	−0.0073 (14)	−0.0039 (13)
C31	0.0267 (16)	0.0272 (15)	0.0348 (16)	−0.0034 (13)	0.0022 (13)	0.0112 (13)

### Geometric parameters (Å, °)

**Table:**

O7—C8	1.383 (3)	C14—C19	1.530 (3)
O7—C1	1.403 (3)	C14—H14	1.0000
O9—C8	1.192 (3)	C15—C23	1.529 (4)
O13—C12	1.361 (3)	C15—H15	1.0000
O13—C14	1.442 (3)	C17—H17A	0.9800
O16—C15	1.414 (3)	C17—H17B	0.9800
O16—H16	0.85 (4)	C17—H17C	0.9800
O18—C12	1.196 (4)	C21—C28	1.507 (4)
O20—C19	1.321 (3)	C21—C29	1.523 (4)
O20—C21	1.489 (3)	C21—C22	1.524 (4)
O24—C23	1.339 (3)	C22—H22A	0.9800
O24—C25	1.499 (3)	C22—H22B	0.9800
O27—C19	1.210 (3)	C22—H22C	0.9800
O30—C23	1.198 (3)	C25—C31	1.519 (4)
C1—C6	1.377 (4)	C25—C26	1.522 (4)
C1—C2	1.392 (4)	C25—C30	1.523 (4)
C2—C3	1.383 (4)	C26—H26A	0.9800
C2—H2	0.9500	C26—H26B	0.9800
C3—C4	1.400 (4)	C26—H26C	0.9800
C3—H3	0.9500	C28—H28A	0.9800
C4—C5	1.403 (4)	C28—H28B	0.9800
C4—C10	1.464 (4)	C28—H28C	0.9800
C5—C6	1.387 (4)	C29—H29A	0.9800
C5—H5	0.9500	C29—H29B	0.9800
C6—H6	0.9500	C29—H29C	0.9800
C8—C17	1.490 (4)	C31—H31A	0.9800
C10—C11	1.331 (4)	C31—H31B	0.9800
C10—H10	0.9500	C31—H31C	0.9800
C11—C12	1.484 (4)	C30—H30A	0.9800
C11—H11	0.9500	C30—H30B	0.9800
C14—C15	1.523 (4)	C30—H30C	0.9800
			
C8—O7—C1	116.5 (2)	O27—C19—C14	120.4 (2)
C12—O13—C14	116.1 (2)	O20—C19—C14	112.6 (2)
C15—O16—H16	113 (3)	O20—C21—C28	110.3 (2)
C19—O20—C21	120.7 (2)	O20—C21—C29	109.5 (2)
C23—O24—C25	120.0 (2)	C28—C21—C29	113.5 (3)
C6—C1—C2	121.7 (3)	O20—C21—C22	101.4 (2)
C6—C1—O7	118.3 (3)	C28—C21—C22	110.8 (3)
C2—C1—O7	119.9 (3)	C29—C21—C22	110.8 (2)
C3—C2—C1	118.7 (3)	C21—C22—H22A	109.5
C3—C2—H2	120.6	C21—C22—H22B	109.5
C1—C2—H2	120.6	H22A—C22—H22B	109.5
C2—C3—C4	121.0 (3)	C21—C22—H22C	109.5
C2—C3—H3	119.5	H22A—C22—H22C	109.5
C4—C3—H3	119.5	H22B—C22—H22C	109.5
C3—C4—C5	118.6 (3)	O30—C23—O24	127.7 (3)
C3—C4—C10	123.6 (3)	O30—C23—C15	122.7 (3)
C5—C4—C10	117.6 (3)	O24—C23—C15	109.6 (2)
C6—C5—C4	120.7 (3)	O24—C25—C31	109.1 (2)
C6—C5—H5	119.6	O24—C25—C26	102.0 (2)
C4—C5—H5	119.6	C31—C25—C26	111.2 (2)
C1—C6—C5	119.2 (3)	O24—C25—C30	108.9 (2)
C1—C6—H6	120.4	C31—C25—C30	113.5 (3)
C5—C6—H6	120.4	C26—C25—C30	111.5 (3)
O9—C8—O7	122.3 (3)	C25—C26—H26A	109.5
O9—C8—C17	127.4 (3)	C25—C26—H26B	109.5
O7—C8—C17	110.2 (2)	H26A—C26—H26B	109.5
C11—C10—C4	128.2 (3)	C25—C26—H26C	109.5
C11—C10—H10	115.9	H26A—C26—H26C	109.5
C4—C10—H10	115.9	H26B—C26—H26C	109.5
C10—C11—C12	118.1 (3)	C21—C28—H28A	109.5
C10—C11—H11	120.9	C21—C28—H28B	109.5
C12—C11—H11	120.9	H28A—C28—H28B	109.5
O18—C12—O13	123.5 (3)	C21—C28—H28C	109.5
O18—C12—C11	126.5 (3)	H28A—C28—H28C	109.5
O13—C12—C11	110.1 (2)	H28B—C28—H28C	109.5
O13—C14—C15	107.1 (2)	C21—C29—H29A	109.5
O13—C14—C19	112.6 (2)	C21—C29—H29B	109.5
C15—C14—C19	109.2 (2)	H29A—C29—H29B	109.5
O13—C14—H14	109.3	C21—C29—H29C	109.5
C15—C14—H14	109.3	H29A—C29—H29C	109.5
C19—C14—H14	109.3	H29B—C29—H29C	109.5
O16—C15—C14	111.6 (2)	C25—C31—H31A	109.5
O16—C15—C23	110.2 (2)	C25—C31—H31B	109.5
C14—C15—C23	109.4 (2)	H31A—C31—H31B	109.5
O16—C15—H15	108.5	C25—C31—H31C	109.5
C14—C15—H15	108.5	H31A—C31—H31C	109.5
C23—C15—H15	108.5	H31B—C31—H31C	109.5
C8—C17—H17A	109.5	C25—C30—H30A	109.5
C8—C17—H17B	109.5	C25—C30—H30B	109.5
H17A—C17—H17B	109.5	H30A—C30—H30B	109.5
C8—C17—H17C	109.5	C25—C30—H30C	109.5
H17A—C17—H17C	109.5	H30A—C30—H30C	109.5
H17B—C17—H17C	109.5	H30B—C30—H30C	109.5
O27—C19—O20	126.9 (2)		
			
C8—O7—C1—C6	76.9 (3)	O13—C14—C15—O16	−72.5 (3)
C8—O7—C1—C2	−106.7 (3)	C19—C14—C15—O16	49.7 (3)
C6—C1—C2—C3	1.5 (4)	O13—C14—C15—C23	49.7 (3)
O7—C1—C2—C3	−174.9 (3)	C19—C14—C15—C23	171.9 (2)
C1—C2—C3—C4	0.6 (4)	C21—O20—C19—O27	1.7 (4)
C2—C3—C4—C5	−1.7 (4)	C21—O20—C19—C14	−175.0 (2)
C2—C3—C4—C10	172.4 (3)	O13—C14—C19—O27	172.2 (2)
C3—C4—C5—C6	0.8 (4)	C15—C14—C19—O27	53.4 (3)
C10—C4—C5—C6	−173.6 (3)	O13—C14—C19—O20	−10.8 (3)
C2—C1—C6—C5	−2.3 (4)	C15—C14—C19—O20	−129.6 (2)
O7—C1—C6—C5	174.1 (2)	C19—O20—C21—C28	60.5 (3)
C4—C5—C6—C1	1.1 (4)	C19—O20—C21—C29	−65.0 (3)
C1—O7—C8—O9	12.1 (4)	C19—O20—C21—C22	177.9 (2)
C1—O7—C8—C17	−167.0 (3)	C25—O24—C23—O30	0.5 (4)
C3—C4—C10—C11	10.1 (5)	C25—O24—C23—C15	−178.7 (2)
C5—C4—C10—C11	−175.8 (3)	O16—C15—C23—O30	8.7 (4)
C4—C10—C11—C12	−175.2 (3)	C14—C15—C23—O30	−114.4 (3)
C14—O13—C12—O18	−2.7 (4)	O16—C15—C23—O24	−172.1 (2)
C14—O13—C12—C11	177.6 (2)	C14—C15—C23—O24	64.8 (3)
C10—C11—C12—O18	7.1 (4)	C23—O24—C25—C31	−60.6 (3)
C10—C11—C12—O13	−173.3 (2)	C23—O24—C25—C26	−178.3 (2)
C12—O13—C14—C15	−157.2 (2)	C23—O24—C25—C30	63.8 (3)
C12—O13—C14—C19	82.8 (3)		

### Hydrogen-bond geometry (Å, °)

**Table:**

*D*—H···*A*	*D*—H	H···*A*	*D*···*A*	*D*—H···*A*
O16—H16···O27^i^	0.85 (4)	2.06 (4)	2.842 (3)	153 (4)
O16—H16···O30	0.85 (4)	2.26 (4)	2.688 (3)	111 (3)

Symmetry codes: (i) *x*−1, *y*, *z*.
